# Influence of pre-processing criteria on analysis of accelerometry-based physical activity

**DOI:** 10.1371/journal.pone.0316357

**Published:** 2025-01-02

**Authors:** Bing Han, Lilian Perez, Deborah A. Cohen, Rachana Seelam, Kathryn P. Derose

**Affiliations:** 1 Kaiser Permanente Southern California, Pasadena, CA, United States of America; 2 RAND Corporation, Santa Monica, CA, United States of America; 3 Department of Health Promotion and Policy, University of Massachusetts Amherst, Amherst, MA, United States of America; Universiti Malaya, MALAYSIA

## Abstract

**Background:**

Accelerometers are widely adopted for physical activity (PA) measurement. Accelerometry data require pre-processing before entering formal statistical analyses. Many pre-processing criteria may influence PA outcomes and the processed sample, impacting results in subsequent statistical analyses.

**Aim:**

To study the implications of pre-processing criteria for accelerometer data on outputs of interest in physical activity studies.

**Methods:**

We used the ActiGraph hip-worn accelerometry data from 538 adult Latino participants. We studied four most important domains of pre-processing criteria (wear-time, minimum wear-time, intensity level, and modified bouts). We examined the true sample size in pre-processed data, the moderate-to-vigorous physical activity (MVPA) outcome, and regression coefficients of age and gender predicting MVPA.

**Results:**

Many pre-processing criteria have minimum impact to the output of interest. However, requirements for minimum wear-time can have high influence on subsequent analyses for MVPA. High requirements for wear-time (e.g., minimum of 5 days with more than 12 hours of wear-time per day) lead to weakened statistical efficiency in estimating the relationship between potential predictors and the MVPA outcome. Intensity levels using vector magnitude triaxial counts yielded drastically different results than those using conventional vertical axis counts.

**Conclusion:**

Moderate changes in minimum wear-time can yield notably different output data and subsequently influence analyses assessing the impacts of interventions on MVPA behaviors. Processed data using vector magnitude and conventional vertical axis counts are not directly comparable. Sensitivity analyses using alternative pre-processing scenarios are highly recommended to verify the robustness of analyses for accelerometry data.

## Introduction

Physical inactivity increases the risk and severity of multiple chronic diseases and is the underlying cause of over 10% of deaths in the United States [1, 2]. Accelerometers are widely used in research to measure human physical activity (PA) and are typically worn on the hip or the wrist [3]. Compared with other objective measurement instruments including lab-based techniques and direct observations, accelerometers are self-administered, more economical, scalable, and suitable for studying a large population longitudinally [4–9]. These unique features make accelerometers the preferred measurement instrument in many studies aimed to quantify individual-level PA behavior and to evaluate the effects of PA interventions. Accelerometers have been used as the gold standard to calibrate the less accurate self-reported PA measures [4, 5, 10].

Count-based accelerometry data are in the format of counts at the epoch level, where each epoch is usually 30 seconds or one minute, for the entire duration the accelerometer has been operating. The count data can contain many zeros, very low values, and erratic values because either the device has not been worn or the wearer has been sedentary or did not wear correctly. The count-based accelerometer data must use a pre-processing procedure to eliminate invalid or meaningless (i.e., non-wear) epochs and to convert counts to more interpretable outcomes, such as time spent in a certain PA intensity level.

The pre-processing procedure for count-based accelerometer data includes multiple steps: differentiating wear-time from non-wear-time; imposing minimum requirements for wear-time; defining PA intensity levels, in particular, the cutoff for moderate-to-vigorous physical activity (MVPA); and clustering PA in modified bouts. Each step consists of further technical details with alternative specifications [11]. Past health and behavioral studies using accelerometer data have not always provided details of their pre-processing steps. Many studies simply conformed to a convention adopted in prior studies [12].

The impact of the pre-processing procedure for count-based accelerometer data has been noted and investigated in the literature with mixed findings. Several studies acknowledged that choices of pre-processing criteria may influence statistical analyses further downstream [12–16]. For example, a recent study reported that by altering the MVPA intensity cutoff and using bouts vs. all minutes, the sample proportion meeting the national PA guidelines varied dramatically from 3% to 96% [17]. Variations in pre-processing criteria, such as wear-time validation algorithms, minimum requirement for wear-time, and cutoffs for PA levels, can lead to significant changes in both the mean time and the percent of time in every PA levels [15, 18, 19]. A prior study developed empirical approximation formulas for the MVPA outcome by epoch length and cutoff points with and without bouts [20]. Another study showed that different pre-processing criteria altered the significance level of the relationship between a socio-economic predictor and PA outcome, specifically from statistically significant (*p* < .001) to non-significant (*p*>.05) [14]. This last study is particularly concerning since many health studies aimed to estimate the relationship between predictors (e.g., a person-level characteristics, a group-level environmental factor, exposure to an intervention, etc.) and the MVPA outcome, rather than the absolute level of the MVPA outcome. This prior evidence suggests that pre-processing criteria can fundamentally influence the findings in such type of health studies. Contrary to these studies above, a study reported that various pre-processing criteria did not alter the findings of PA patterns among obese diabetic patients but only changed the sample size by about 10%, i.e., participants that did not meet the selected criteria were dropped from analyses [21]. Prior studies also have mixed views on using the vector magnitude using triaxial counts. While one study praised the vector triaxial counts for improving identification of valid wear-time compared with the standard vertical axis counts [22], another study did not find that the vector magnitude with triaxial counts can further improve predictive performance of counts for energy expenditure beyond using the vertical axis counts only [23]. Several studies warned to not directly compare data in vector magnitude across different types of accelerometers [24, 25].

In this paper, we aim to investigate multiple important pre-processing criteria by a comprehensive study design. We study the true sample size in pre-processed data, the absolute level of the MVPA outcome, and regression coefficients of age and gender predicting MVPA. The present study describes a systematic method that can be adopted by other researchers as a sensitivity analysis to guide decisions for pre-processing and analyzing accelerometer data. Our findings can also provide useful reference for future health studies using accelerometer to set up their pre-processing procedures.

## Method

### Ethics statement

We used primary data collected from an ongoing cluster randomized controlled trial, i.e., the parent study. The parent study was approved by the Human Subjects Protection Committee (Assurance No. FWA00003425, IRB No. IRB00000051 [2018]), which is the institutional review board to review research involving human subjects at the RAND Corporation. The parent study was registered in ClinicalTrials.gov (NCT03858868). The goal of the parent study is to promote PA among adult churchgoing Latinos from six churches in predominantly-Latino neighborhoods in and near East Los Angeles, California [26, 27]. All study participants were adult, from whom written consents were collected before data collection.

### Sample and data

Potentially eligible participants from the six participating churches were screened for a history and symptoms of health conditions that could preclude PA [28]. Upon consent, participants eligible for the trial were asked to wear an ActiGraph wGT3X-BT activity monitor around their hip for a week, removing it only during water activities (e.g., showering) and when sleeping. To reduce the likelihood of draining batteries in the field, the accelerometers were set with the sampling rate of 30 Hz, the sleep mode enabled, and the inertial measurement unit sensor disabled. The low frequence extension option was not used since the parent study is focused on MVPA. The primary data consisted of 538 individuals and data were collected in 2019. Table 1 describes the characteristics of the sample. We processed the data using ActiLife software v6.13.4 (ActiGraph, Pensacola, FL) and analyzed data using SAS 9.4 and R 4.1.

**Table 1:** Characteristics of study participants (n = 538).

Characteristic	Mean (standard deviation) or percentages
Age in years	51.9 (14.0)
Gender: female	77.6%
Education: high school or higher	40.7%
Self-rated English proficiency: good or above	39.2%
Marital status	
Single, never married	21.7%
Married or living with partner	58.2%
Other statuses	20.1%
Lived in the U.S. for the whole life	26.0%
Body mass index	31.8 (7.3)
# days with any accelerometry data	9.3 (6.4)

### Detailed steps in a pre-processing process

A pre-processing procedure can be implemented in a general-purpose statistical software package such as SAS and R. Researchers of ActiGraph accelerometers often use the software ActiLife specialized for pre-processing and analyzing ActiGraph accelerometry data. In this paper we worked with the ActiLife software tool. A pre-processing process can involve many steps and details. In this paper we selected the most important or required steps to study. We used the following key terms to organize these steps in line with the interface in ActiLife: *domain*, *criterion*, and *option*. From the top, a processing procedure is split into four sequential domains, where each domain has a distinct task: validation domain 1 (to find valid wear-time), validation domain 2 (to apply minimum valid wear-time requirement), scoring domain 1 (to implement MVPA intensity thresholds), and scoring domain 2 (to identify modified bouts by MVPA intensity levels). Each domain consists of multiple criteria, where a criterion is a specific decision users need to make. A criterion usually has two or more options, from which the user must choose one. Most criteria have one option set as default in ActiLife. Table 2 lists all domains, criteria, and options under consideration in this paper, which are used throughout the remainder of this paper. Lastly, we further define the term *scenario*: a scenario is a unique set of options selected across all criteria of the four domains. Two scenarios are different if at least one criterion has different options selected between the two scenarios.

**Table 2:** Domains, criteria, and options for pre-processing accelerometer data considered in this paper.

Domains	Criteria	Options
Validation domain 1 (valid wear-time)	Algorithm	• *Troiano_2007*: non-wear period is broken by any minute > 100 counts, or 2 consecutive minutes > 0 counts •*Choi_2011*: non-wear period is broken by 2 consecutive minutes > 0 counts, or two spikes (< 2 minutes with >0 counts) within 30 minutes to each other.
	Vector magnitude	• *No*: default on the vertical axis •*Yes*: Euclidean metric combining all three axis.
Validation domain 2 (minimum wear-time)	Minimum wear-time for a valid day	A valid day should have at least 6, 8, 10, or 12 valid wear hours.
	Minimum # valid days	A participant should have at least 1, 3, or 5 valid days.
	Minimum # valid days during weekends	A participant should have at least 0 or 1 valid day on weekend.
Scoring domain 1 (MVPA threshold)	Thresholds on shocks per minute	• *V_1952*: per minute counts on the vertical axis, sedentary<100, light<1952, moderate<5274, vigorous<9498; very vigorous > 9498 •*V_2020*: per minute counts on the vertical axis, sedentary<100, light<2020, moderate <5998, vigorous > 5998 •*VM3_2690*: this option requires selecting the “vector” option in the vector magnitude criterion. Per minute counts on Euclidean measure, light<2690, moderate < 6167, vigorous<9642, very vigorous >9642
Scoring domain 2 (modified bouts of MVPA)	MVPA bout definition: minimum length is 10 minutes, and 2 minutes out of range allowed within a bout for all options	• *Bout_1952*: vertical count threshold of 1952, same as in *V_1952* •*Bout_2020*: vertical count threshold of 2020, same as in *V_2020* •*Bout_2690*: vector magnitude threshold of 2690, same as in *VM3_2690*

#### Validation domains 1: To find wear-time

Non-wear-time includes periods when the device was not worn by a participant during the indicated timespan. In this paper, we considered two criteria in validation domain 1: an algorithm to identify valid wear-time and the vector magnitude for counts. First, there are two built-in options in ActiLife to identify non-wear-time, referred to as *Troiano_2007* and *Choi_2011* in this paper [29–31]. These two options aim to identify patterns in the count data that match with pre-defined non-wear-time patterns with different algorithms. One notable difference is that *Troiano_2007* defines a non-wear period as at least 60 consecutive minutes of zero count values whereas *Choi_2011* uses a minimum of 90 consecutive minutes. Thus, *Choi_2011* may identify shorter non-wear-time than *Troiano_2007*. The second criterion is referred to as “vector magnitude” with two options. The default option, i.e., “No”, denoted as requests that only the counts on the vertical axis of an accelerometer is used as input data for the selected algorithm above. The alternative option, i.e., “Yes”, uses a Euclidean-type metric combining triaxial counts [11, 23, 24].

#### Validation domains 2: To apply minimum wear-time requirement

A minimum length of wear-time is necessary to reliably assess PA patterns at the daily or person level [29, 32, 33]. First, a “valid day” needs to exceed a minimum wear-time threshold (e.g., 10 hours/day). A more stringent threshold for defining a valid day (e.g., 12 hours/day) will result in fewer valid days and potentially a smaller sample size in the analytic dataset. Next, a study participant may need to have a minimum number of valid days to be included in analysis. This requirement is due to the high inter-day variations in a person’s PA. Researchers may be interested in a person-level outcome, e.g., whether a person meets the national guideline of weekly MVPA time. Ideally, they may wish to have at least one entire week of valid days to assess this outcome for a participant. However, due to participant non-compliance, the actual data collected are usually much less than ideal. The minimum number of valid days (e.g., an entire week) may screen out participants with too few valid days and could potentially bias the sample by only including the most adherent participants. Lastly, the minimum wear-time requirements can also specify a minimum number of valid weekdays and weekend days to capture greater variability in PA levels across the week.

For this domain, we compare three options: minimum time for a valid day (6, 8, 10, 12 hours of valid wear-time), minimum valid days for a participant (1, 3, 5 valid days), and minimum valid days during weekends (0 or 1 valid day), where 0 means no requirement for a valid weekend day.

#### Scoring domain 1: MVPA intensity threshold

After processing the validation domains for valid wear-time and minimum time, the count data are processed to differentiate activity intensity levels, which are more meaningful and interpretable for analyses. Scoring domain 1 contains pre-defined thresholds on accelerometer counts to define intensity levels; we focused on MVPA given this intensity level is associated with multiple health benefits and is the focus of national PA guidelines. The three options to identify adult MVPA are built in ActiLife, referred to as *V_1952*, *V_2020*, and *VM3_2690* hereafter. Specifically, *V_1952* defines threshold for MVPA at 1,952 counts per minute on the vertical axis [34], *V_2020* uses 2,020 counts per minute on the vertical axis [29], and *VM3_2690* defines MVPA as 2,690 counts per minute based on the vector magnitude tri-axial counts [24]. The threshold for *V_2020* is a more stringent requirement than *V_1952*. The *VM3_2690*’s cutoff value is based on vector magnitude counts and not comparable with the other two options that are based only on the vertical axis.

#### Scoring domains 2: Modified bouts

Bouts are a consecutive period of time during which the PA intensity level is consistent. Arguably, PA times accrued by bouts are robust to temporary changes in a person’s PA levels or abrupt measurement errors. While all three pre-processing domains discussed previously are mandatory, this domain is optional depending on one’s interest in bouts. Modified bout definitions can also be customized in ActiLife: length of a bout, the logic to break an ongoing bout, and the MVPA threshold to define a bout. In this paper, we used the default logic to break a bout and the default minimum length of 10 minutes [29]. We considered a single criterion with three options in this domain, corresponding to three options under scoring domain 1. When MVPA intensity levels are defined by a certain threshold, bouts should also be based on the same threshold.

### Statistical analysis design

We tested various pre-processing scenarios using the design of a numerical experiment, where a criterion with multiple options can be seen as a design factor with multiple factor levels in an experiment. The full-factorial experiment design has a total of 864 scenarios by enumerating all combinations of options in all criteria across the four domains. In this paper, we elected to use a partial factorial design with a subset of scenarios. The partial factorial design was preferred for two reasons. First, the empirical evidence from the partial factorial design with a few representative scenarios was sufficiently strong to show the influence of a single criterion. Second, many scenarios in the full factorial design are unrealistic. For example, some conceptual scenarios can use one cutoff for defining MVPA intensity level but a different cutoff for defining MVPA bouts. These counterintuitive scenarios are neither practical nor interpretable. Our partial factorial design selected a few reasonable and representative scenarios. We first studied the influence of validation domain 1 (valid wear-time), by specifying options in the other three domains. Next, we studied the influence of scoring domain 1 and 2 by specifying the options in validation domains 1 and 2. Scoring domains 1 and 2 were studied together, because both scoring domains should always use the same MVPA cutoff values. Lastly, we studied the influence of validation domain 2 (minimum wear-time), by fixing options in the other three domains. Table 3 lists all the selected scenarios in details.

**Table 3:** List of selected scenarios in this paper.

Scenarios	Validation domain 1 (valid wear-time)	Validation domain 2 (minimum wear-time)	Scoring domain 1 (MVPA threshold)	Scoring domain 2 (modified bouts of MVPA)
1	Algorithm: *Troiano_2007* Vector magnitude: No	10 hours/day 3 days/week, no weekend requirements	*V_1952*	*---*
2	Algorithm: *Troiano_2007* Vector magnitude: Yes			
3	Algorithm: *Choi_2011* Vector magnitude: No			
4	Algorithm: *Choi_2011* Vector magnitude: Yes			
5	Algorithm: *Troiano_2007* Vector magnitude: No		*V_1952*	*---*
6			*V_2020*	
7			*VM3_2690*	
8			*---*	*V_1952*
9				*V_2020*
10				*VM3_2690*
11		6 hours/day, 1 day/week, no weekend needed	*V_1952*	*---*
12		6 hours/day, 1 day/week, 1 weekend day		
13		6 hours/day, 3 days/week, no weekend day needed		
14		6 hours/day, 3 days/week, 1 weekend day		
15		6 hours/day, 5 days/week, no weekend day needed		
16		6 hours/day, 5 days/week, 1 weekend day		
17		8 hours/day, 1 day/week, no weekend needed		
18		8 hours/day, 1 day/week, 1 weekend day		
19		8 hours/day, 3 days/week, no weekend day needed		
20		8 hours/day, 3 days/week, 1 weekend day		
21		8 hours/day, 5 days/week, no weekend day needed		
22		8 hours/day, 5 days/week, 1 weekend day		
23		10 hours/day, 1 day/week, no weekend needed		
24		10 hours/day, 1 day/week, 1 weekend day		
25		10 hours/day, 3 days/week, no weekend day needed		
26		10 hours/day, 3 days/week, 1 weekend day		
27		10 hours/day, 5 days/week, no weekend day needed		
28		10 hours/day, 5 days/week, 1 weekend day		
29		12 hours/day, 1 day/week, no weekend needed		
30		12 hours/day, 1 day/week, 1 weekend day		
31		12 hours/day, 3 days/week, no weekend day needed		
32		12 hours/day, 3 days/week, 1 weekend day		
33		12 hours/day, 5 days/week, no weekend day needed		
34		12 hours/day, 5 days/week, 1 weekend day		

### Study outcomes for measuring sensitivity in pre-processing scenarios

To examine the potential influence of different pre-processing scenarios in the analysis of accelerometry data, we examined multiple outcomes. First, we examined changes in sample size after going through the two validation domains. We examined the number of participants with sufficient valid wear days, the total number of valid days, and the average number of valid days per participant, all of which reflect the concept of effective sample size in a hierarchical model.

Second, we examined the absolute MVPA level. This outcome is directly affected by the two scoring domains but may also vary by the two validation domains. For each scenario, we calculated the mean and standard deviation (SD) of the MVPA time at the person-day level. We also calculated the percent of participants meeting national MVPA guidelines of 150 minutes/week by multiplying the daily MVPA by 7 and compared with the guideline. Except for studying scoring domain 2 (modified bouts), the daily MVPA outcome always refers to all time above the selected MVPA threshold. Wherever applicable, we conducted paired t-tests to assess the statistical significance of the difference in daily MVPA between comparable scenarios.

Third, we examined the regression coefficients from a multiple regression model. The model regressed the daily MVPA time versus two predictors: age in years and gender (female versus male), whose coefficient estimates serve as our study outcomes. Other covariates in the regression models included body mass index (BMI) and average wear-time per day. However, these latter two covariates were mostly not significant in the models and excluding them did not affect the findings. We did not further adjust for other possible covariates because we were not interested in the actual estimate of any association but instead to observe their variations by pre-processing scenario.

## Results

### Influence by validation domain 1 (valid wear-time)

Table 4 shows the results for studying the influence of varying validation domain 1 (valid wear-time), holding options of the other three domains constant. The four scenarios give generally similar results. Using the same vector magnitude option, the *Choi_2011* algorithm always yielded a slightly larger sample size compared to using *Troiano_2007*. For example, when not using the vector magnitude option, *Choi_2011* resulted in 449 eligible participants, 2,443 valid person-days, and an average of 5.57 valid days per eligible participants. By contrast, *Troiano_2007* yielded 433 eligible participants, 2,343 valid person-days, and an average of 5.41 valid days per eligible participant. However, within the same algorithm, using the vector magnitude option gives slightly larger sample sizes than using the vertical axis only. For example, using the *Troiano_2007* algorithm, with the vector magnitude, the sample size was 443 eligible participants whereas it was 433 with the vertical axis (similar results observed for valid person-days and average valid days per eligible participant).

**Table 4:** Influence of validation domain 1 (valid wear-time) on study outcomes. MVPA times are all minutes above threshold ^a^.

Algorithm	Vector magnitude	# eligible participants with sufficient data	# valid person-days	Average # valid days per person	Mean daily MVPA time (minutes)	SD of daily MVPA time (minutes)	Regression coefficient: age in years	Regression coefficient women vs. men
*Troiano_2007*	No	433	2343	5.41	26.07	21.06	-0.33***	-10.9***
	Yes	443	2499	5.51	25.65	20.87	-0.33***	-10.0***
*Choi_2011*	No	449	2443	5.57	25.38	20.68	-0.30***	-10.0***
	Yes	452	2529	5.60	25.33	20.71	-0.30***	-10.2***

^a^ Corresponding to scenarios 1–4 in Table 3.

P-value <0.001***,<0.01**, < = 0.05*.

The modest differences in sample sizes by changing the criteria for domain 1 do not yield notable changes in the outcomes of MVPA time (Table 4). The mean daily MVPA time is between 25.66 and 26.07 minutes per day, and the SD of daily MVPA time is between 20.68 and 21.06 minutes. Differences across the four scenarios are very small. Age is consistently negatively related to daily MVPA time: one year increase in age is associated with a reduction of 0.33 minutes in daily MVPA time (*p*<0.001). Women have on average 10 fewer minutes per day of MVPA than men (*p*<0.001).

### Influence by scoring domain 1 (MVPA intensity threshold)

Table 5 shows the three scenarios comparing the influence of varying MVPA intensity threshold when the other criteria are held constant. The *V_2020* option resulted in less average daily MVPA time than *V_1952* (24.35 vs. 26.07 minutes per day, respectively, *p* = 0.03) and a smaller SD (20.24 vs. 21.06 minutes per day, respectively). Consequently, *V_2020* resulted in fewer people meeting the MVPA guideline (46.9%) than *V_1952* (49.9%). *V_2020* also had slightly smaller absolute values in regression coefficients than *V_1952* (coefficients of age: -0.31 vs. -0.33, and coefficients of gender: -10.3 vs. -10.9). Despite the consistent pattern, the differences between *V_1952* and *V_2020* in our study outcomes seem to be small or very small.

**Table 5:** Influence of scoring domain 1 (MVPA intensity threshold) on study outcomes. MVPA times are all minutes above threshold. (n = 433 eligible patients, 2,343 valid person-days) ^a^.

Options	Average daily MVPA time (minutes)	SD of daily MVPA time (minutes)	Beta: age in years (SE)	Beta: female-male	% meeting MVPA guidelines
*V_1952*	26.07	21.06	-0.33***	-10.9***	49.9
*V_2020*	24.35	20.24	-0.31***	-10.3***	46.9
*VM3_2690*	45.21	33.69	-0.59***	-20.73***	74.7

^a^ Corresponding to scenarios 5–7 in Table 3.

P-value <0.001***, <0.01**, < = 0.05*.

The *VM3_2690* option, based on the vector magnitude, yielded very different results than the two options using vertical axis counts. Compared with *V_1952* and *V_2020*, the mean and SD of daily MVPA time increased from 60 to 80% (20 more minutes per day in sample mean and 12 minutes per day in SD, approximately). Consequently, approximately an additional 25% of participants were deemed as meeting the national MVPA guidelines. The absolute values of regression coefficients of age and gender were also nearly doubled, compared with the previous two options.

### Influence by scoring domain 2 (modified bouts)

Table 5 shows three scenarios for MVPA time by modified bouts. These three scenarios are the same as in Table 5, except that the MVPA outcomes are based on 10-minute bouts rather than using all minutes. Table 6 shows a pattern similar to Table 5. The two options using vertical axis counts, *Bout_1952* and *Bout_2020*, produced almost identical MVPA outcomes and regression coefficients. *Bout_2690* using vector magnitude had much larger mean and SD in daily MVPA time, as well as substantially larger regression coefficients in absolute values. Notably, the two options using vertical axis counts did not have significant regression coefficients (*p*>0.05), which indicates a great reduction of statistical power.

**Table 6:** Influence of scoring domain 2 (modified bouts) on study outcomes (n = 433 patients, 2,343 person-days) ^a^.

Threshold options	Average daily MVPA time (minutes in modified bout)	SD of daily MVPA time (minutes in modified bout)	Beta: age in years (SE)	Beta: female-male	% meeting MVPA guidelines
*Bout_1952*	9.36	13.51	-0.09	-1.81	14.0
*Bout_2020*	9.36	13.51	-0.09	-1.81	14.0
*Bout_2690*	14.49	19.12	-0.16*	-6.72**	24.8

^a^ Corresponding to scenarios 8–10 in Table 3.

P-value <0.001***, <0.01**, < = 0.05*.

### Influence by validation domain 2 (minimum wear-time)

**Fig 1:**
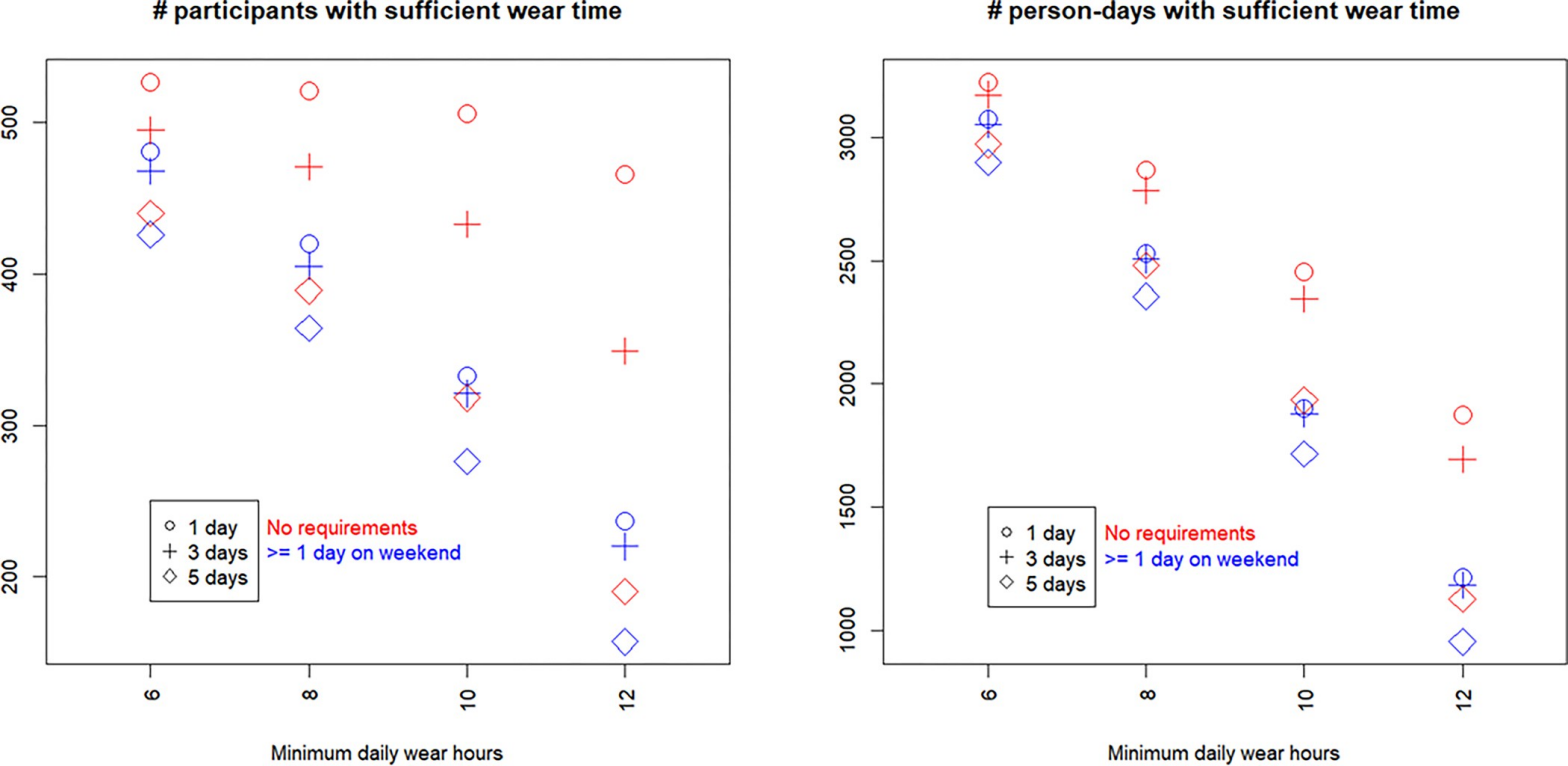
Influence of validation domain 2 (minimum time) on sample sizes. x-axis marks the four levels of minimum wear-time per day, legends mark the three numbers of valid days, and colors differentiate requirements for valid day on weekends. These are scenarios 11–34 in Table 3.

**Fig 2:**
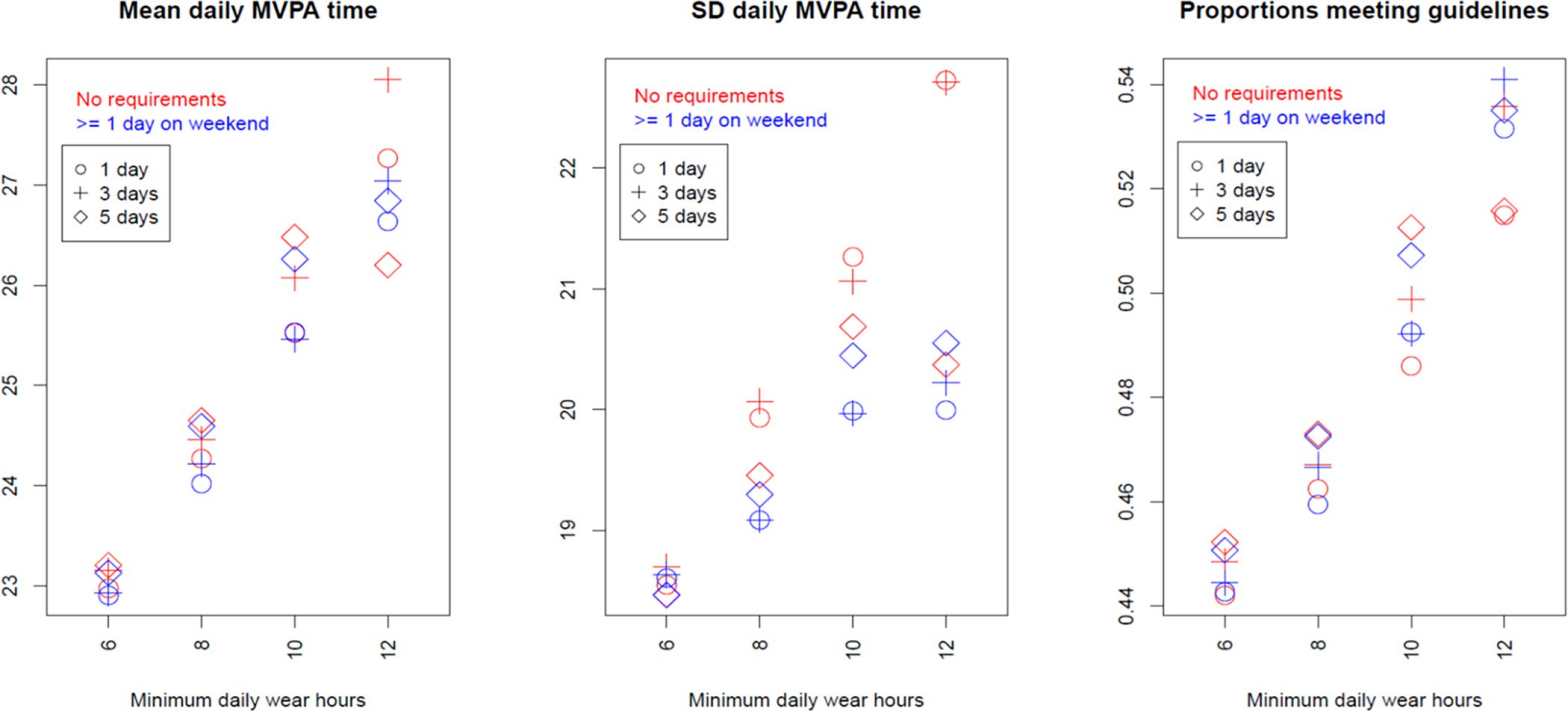
Influence of validation domain 2 (minimum time) on MVPA outcomes. x-axis marks the four levels of minimum wear-time per day, legends mark the three levels of minimum valid days, and colors differentiate requirements for valid weekend day. These are scenarios 11–34 in Table 3.

**Fig 3:**
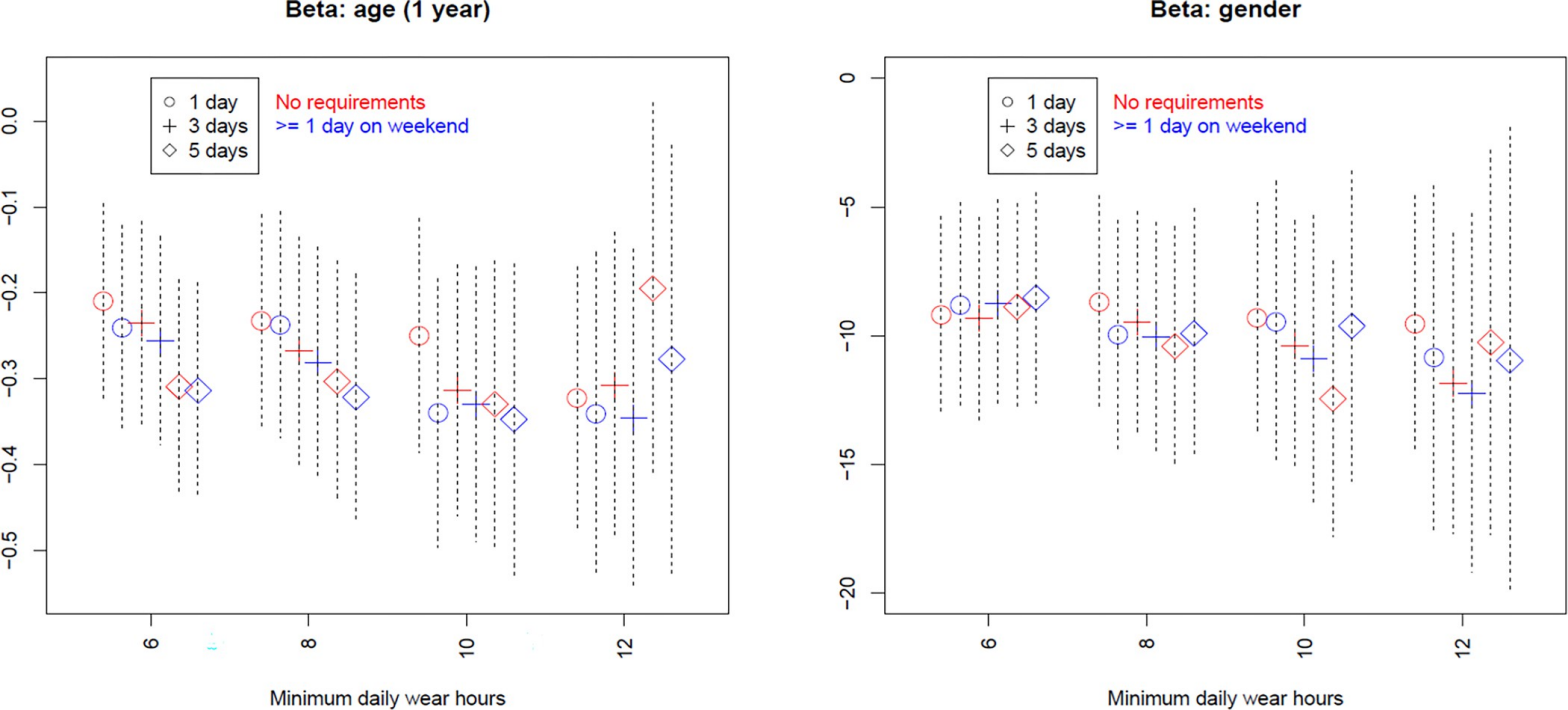
Influence of validation domain 2 (minimum time) on regression coefficients. x-axis marks the four levels of minimum wear-time per day, legends mark the three levels of minimum valid days, and colors differentiate requirements for valid weekend day. Dashed bars are 95% CI. These are scenarios 11–34 in Table 3.

Figs 1–3 present the results of the 24 scenarios studying the influence of varying the validation domain 2 (minimum wear-time), when other criteria were held constant.

Fig 1 shows that using more stringent requirements—i.e., longer minimum wear-time per day, more total valid days, or at least one valid weekend day—resulted in substantially smaller sample sizes than options with looser requirements. The number of eligible participants ranged between 157 (with the strictest requirements) and 557 (with the loosest requirements) and the number of valid person-days ranged between 953 (strictest) and 3,225 (loosest). Not only was the range sizable, but even a seemingly small change in a pre-processing option appeared to greatly alter the sample size. For example, when requiring 12 hours of wear-time and 3 valid days, further requiring at least one valid day on weekends reduced the sample sizes from 349 participants with 1,694 valid person-days to 220 participants with 1,183 valid person-days. When requiring 5 valid days per participant and no requirements on weekends, changing from 10 hours of minimum daily wear-time to 12 hours reduced the sample size from 318 participants and 1,933 valid person-days to 190 participants with 1,127 valid person-days.

Fig 2 shows that the daily MVPA time outcome differed notably across the 24 potential scenarios. Higher minimum wear-time requirements resulted in higher mean daily MVPA times. The SD of daily MVPA time varied moderately across scenarios with a less clear pattern. Based on a crude SD of 20 minutes per day, differences in mean daily MVPA time between any two scenarios had a standardized effect size of 0 to .25 times SD. The proportions meeting national guidelines were higher in most stringent scenario (54.1%) than the least stringent (44.2%).

Fig 3 shows that the inference precision, e.g., the width of the 95% confidence interval (CI), differed substantially across scenarios, which was unsurprising given the largely different sample sizes across scenarios (Fig 1). Point estimates for age as a predictor displayed a moderate level of variation across scenarios. By contrast, point estimates for gender, a relatively stronger predictor than age, showed a small level of variation across scenarios. Significance levels for both predictors varied across scenarios. For example, the association with age was highly significant (*p*<0.001) in all scenarios requiring 10 hours minimum wear-time per day but non-significant (*p*>0.05) in the scenario requiring 12 hours minimum wear-time per day and at least 5 days with no requirements on weekends. Generally, the relatively stringent scenarios had wider CI but often bigger effect sizes, the latter partially compensating the loss of precision, compared with less stringent scenarios.

## Discussion

Our findings highlight the presence and the lack of influence of various pre-processing criteria on accelerometry-assessed PA outcomes. Our study is also among the first to investigate this topic in a community sample of Latino adults. Our main findings include the following. First, numeric algorithms and the decision to use the vector magnitude for identifying valid wear-time did not seem to result in notable influences on all study outcomes. Second, our data suggests that MVPA intensity thresholds based on vertical axis and tri-axial counts are not comparable, although each set of thresholds was independently validated in the literature. The prevalence of meeting PA guidelines varied greatly from very low to moderately high depending on the pre-processing scenarios, which is consistent with prior reports [8, 17]. This finding is concerning since meeting PA guidelines is an important health outcome. Lastly, the requirement for minimum wear-time played an important role influencing downstream statistical analyses. Key take-aways from our analyses are that 1) minimum wear-time requirements are the most influential criteria and thus need to be reported clearly in the methods of research studies; 2) predictors with weak to moderate levels of association with MVPA outcomes, such as age, may be more influenced by the pre-processing criteria than those with strong associations, such as gender. This finding is in agreement with prior studies on the importance of wear-time requirements [18, 19]. Therefore, high requirements for wear-time are not always helpful in studying the associations between MVPA and predictors.

Given these lessons learned, sensitivity analyses based on alternative pre-processing criteria, in particular, minimum wear-time requirement settings, are highly recommended to check the robustness of the subsequent statistical estimates based on pre-processed data. Although such sensitivity analysis cannot establish the validity for any pre-processing criteria, it is informative in the sense of revealing the extent to which the subsequent statistical estimates depend on specific pre-processing choices. For example, in an imaginary study for the association between the MVPA outcome and a particular predictor, the estimated relationship changes its significance level between alternative pre-processing criteria (cf. Fig 3). The presence of this level of sensitivity should be reported as a limitation of the study. Vice versa, if the estimates remain relatively stable between alternative pre-processing criteria, the robustness of results may be claimed as a strength.

It is also worth noting key differences when using modified bouts versus not using them. Since modified bouts are a more stringent requirement to define MVPA than using all available valid wear-time, this resulted in lower MVPA. Our study suggests that MVPA outcomes based on unbouted versus bouted data are essentially two distinct concepts, each of which have their own absolute levels, SD, and associations with potential predictors. Their numeric values, statistical inference, and interpretations are not comparable, even when other pre-processing criteria are the same.

This study is subject to several limitations. First, analyses were based on a single and unique primary dataset. The sampled participants are based on an understudied population and results should be interpreted with caution. The primary data were collected by study participants’ compliance to receive, wear, and return the accelerometer devices. Failure to comply with the basic protocol (e.g., worn by more than one individual) can result in biases in the data. Second, we only used one type of accelerometer with one wear position (hip) and epoch length of 30 seconds. We were not able to study sensitivity due to different wearing position or different epoch lengths. Third, pre-processing was conducted in the proprietary software ActiLife. Despite its powerful functionality, friendly user interface, and popularity among various users, ActiLife is specialized and does not have full data processing capacity. Certain technical issues can be better addressed in a general-purpose statistical software, such as the GGIR library in R. Fourth, pre-processing criteria, in particular, minimum wear-time, can impact measurements of sedentary behavior and light PA measurements, just as they do on measuring MVPA. However, the cutoff for light PA is less well-established compared with the MVPA cutoff. The lower bound for sedentary behavior also overlaps with the algorithm for assessing valid wear-time. These important issues are beyond the scope of the current paper. Future studies are still needed to continue the investigation.

Despite these limitations, our results demonstrate that all pre-processing criteria used in analyzing accelerometry data need to be carefully considered, well understood, and well documented. Certain criteria may be preferred to alternative criteria for some scientific or other reasons. For example, one may prefer *Choi_2011* to *Troiano_2007* for the latter algorithm’s more comprehensive scan over time. Another example is the wear-time requirement of 12 hours for three days, which was used in the widely cited analyses for NHANES accelerometry data. Some may argue all time above threshold makes more sense than time in bouts. While we are not positioned to judge which criteria are more scientifically sound, it is clear that when comparing a study’s results with prior studies, one must be sure to only compare results generated by the same or very similar pre-processing criteria. Results from studies with very different pre-processing procedures are incomparable; improper comparisons can contribute to misleading conclusions. Sensitivity analyses are necessary to check the influence of selected and alternative pre-processing criteria in a study’s main findings. Before presenting any statistical estimates, specific pre-processing criteria that led to the conclusions need to be clearly presented.

## Conclusion

Pre-processing steps for accelerometer data can influence the effective sample size and the magnitude of the MVPA outcome as well as its association with other predictors. Moderate changes in minimum wear-time can yield notably different output data and subsequently influence analyses assessing the impacts of interventions on MVPA behaviors. Processed data using vector triaxial magnitude and conventional vertical axis counts are not directly comparable. Sensitivity analyses using alternative pre-processing scenarios are highly recommended to verify the robustness of analyses for accelerometry data. Between-study comparisons should be based on the same or very similar choices in pre-processing criteria.

## Supporting information

S1 File Deidentified pre-processed analytic data are included in the file “S1 File.zip”. (ZIP)
